# The transverse and vertical distribution of prostate cancer in biopsy and radical prostatectomy specimens

**DOI:** 10.1186/s12885-018-5124-9

**Published:** 2018-12-04

**Authors:** Zhipeng Mai, Zhien Zhou, Weigang Yan, Yu Xiao, Yi Zhou, Zhiyong Liang, Zhigang Ji, Hanzhong Li

**Affiliations:** 1 Department of Urology, Peking Union Medical College Hospital, Chinese Academy of Medical Sciences, 1 WangfujingShuaifuyuan, Beijing, 100730 China; 2 Department of Pathology, Peking Union Medical College Hospital, Chinese Academy of Medical Sciences, 1 WangfujingShuaifuyuan, Beijing, 100730 China

**Keywords:** Spatial distribution, Biopsy, Radical prostatectomy, Apex

## Abstract

**Background:**

Prostate biopsy is the most common method for the diagnosis of prostate cancer and the basis for further treatment. Confirmation using radical prostatectomy specimens is the most reliable method for verifying the accuracy of template-guided transperineal prostate biopsy. The study aimed to reveal the spatial distribution of prostate cancer in template-guided transperineal saturation biopsy and radical prostatectomy specimens.

**Methods:**

Between December 2012 to December 2016, 171 patients were diagnosed with prostate cancer via template-guided transperineal prostate biopsy and subsequently underwent laparoscopic radical prostatectomy. The spatial distributions of prostate cancer were analyzed and the consistency of the tumor distribution between biopsy and radical prostatectomy specimens were compared.

**Results:**

The positive rate of biopsy in the apex region was significantly higher than that of the other biopsy regions (43% vs 28%, *P* < 0.01). In radical prostatectomy specimens, the positive rate was highest at the region 0.9–1.3 cm above the apex, and it had a tendency to decrease towards the base. There was a significant difference in the positive rate between the cephalic and caudal half of the prostate (68% vs 99%, *P* < 0.01). There were no significant differences between the anterior and posterior zones for either biopsy or radical prostatectomy specimens.

**Conclusion:**

The tumor spatial distribution generated by template-guided transperineal prostate biopsy was consistent with that of radical prostatectomy specimens in general. The positive rate was consistent between anterior and posterior zones. The caudal half of the prostate, especially the vicinity of the apex, was the frequently occurred site of the tumor.

## Background

Prostate biopsy is the most common method for the diagnosis of prostate cancer and the basis for further treatment. The application of focal therapy requires higher prostate biopsy accuracy, not only on the positive rate but also on the location of the prostate cancer [1]. A specific and comprehensive spatial distribution of prostate cancer is important for the guidance of prostate biopsy.

Transrectal is currently the primary method for prostate biopsy. However, this approach is associated with several drawbacks, including a relatively high false-negative and infection rate, underestimation of the risk stratification of prostate cancer, and inaccurate estimation of the spatial distribution of prostate cancer with a low detection rate in the anterior and apex zones [2, 3]. With the help of template, transperineal biopsy puncture tissue parallelly to the urethra in a planned way to effectively sample the anterior and apical parts of the prostate and detect small volume lesions. Transperineal template-guided mapping biopsy is considered as the acceptable method for preoperative evaluation of focal therapy [1]. Therefore, it is of great significance for the improvement of the biopsy technique and the selection of post-biopsy treatment plans to specify the spatial distribution of prostate cancer on radical prostatectomy specimens diagnosed by template-guided transperineal biopsy.

Over the past 10 years, we have completed more than 5000 cases of template-guided transperineal prostate biopsies and proposed our own perspective regarding the spatial distribution of prostate cancer on prostate biopsy [4–6]. Confirmation using radical prostatectomy specimens is the most reliable method for verifying the accuracy of template-guided transperineal prostate biopsy [7]. This study was undertaken to reveal the spatial distribution of prostate cancer in template-guided transperineal saturation biopsy and radical prostatectomy specimens.

## Methods

### Patient selection

This study was approved by the ethics committee of Peking Union Medical College Hospital, Chinese Academy of Medical Sciences. Written informed consent was obtained from all subjects. All methods were performed in accordance with the relevant guidelines and regulations. From December 2012 to December 2016, 171 patients of prostate cancer were confirmed by template-guided transperineal saturation biopsy and underwent laparoscopic radical prostatectomy, which was performed by the same surgeon. The inclusion criteria included (1) being diagnosed with prostate cancer during the first transperineal biopsy, (2) having no local lymph node metastasis or distant metastasis and a clinical stage of T1c–T3a, (3) having no endocrine, radio-, or chemotherapy, and (4) having no history of transurethral resection of the prostate.

### Biopsy procedure

**Fig. 1:**
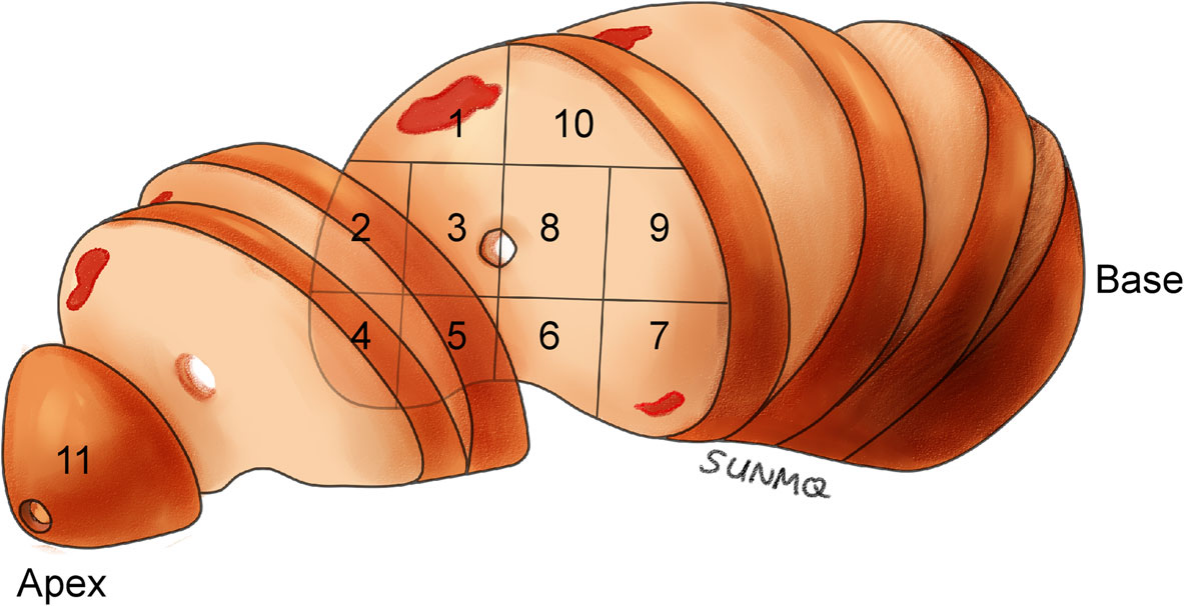
Diagram showing the procedures used for all whole-mount slices from one prostate specimen and the corresponding 11-region map determined by biopsy. The inferior-most 0.5 cm portion of the gland was cut off as the apex. Next, along the cutting edge, whole-mount slices were obtained at 0.4 cm intervals. The remaining portion located approximately 1 cm from the superior-most part of the gland was classified as the base. In the 11-region map, region 11 represented the apex by biopsy, and regions 1–10 represented the mid-gland and the base

A systematic transperineal template-guided 11-region saturation biopsy was adopted [4–6]. The distribution of 11 regions is shown in Fig. 1. A Bard biopsy gun (C.R. Bard; Covington, GA) and an 18-gauge biopsy needle were used. The sampling length was 18 mm.

### Radical prostatectomy specimens

Radical prostatectomy specimens were fixed in 10% neutral-buffered formalin for 48 h. The specimen surface was inked with blue color to evaluate the surgical margin. The apex of the prostate was defined as the inferior-most 0.5 cm portion of the gland. The base of the prostate was defined as the superior-most 1 cm portion of the gland, and the rest was defined as the mid-gland [8]. The general slicing method is shown in Fig. 1. The apex and the base of the prostate were divided into small slices (approximately 0.2 cm) perpendicular to the surface of the gland and were sent for pathological examination together with whole-mount slices of the mid-gland. The prostate was divided by the urethra into anterior and posterior zones or left and right sides in radical prostatectomy specimens and prostate biopsy.

**Fig. 2:**
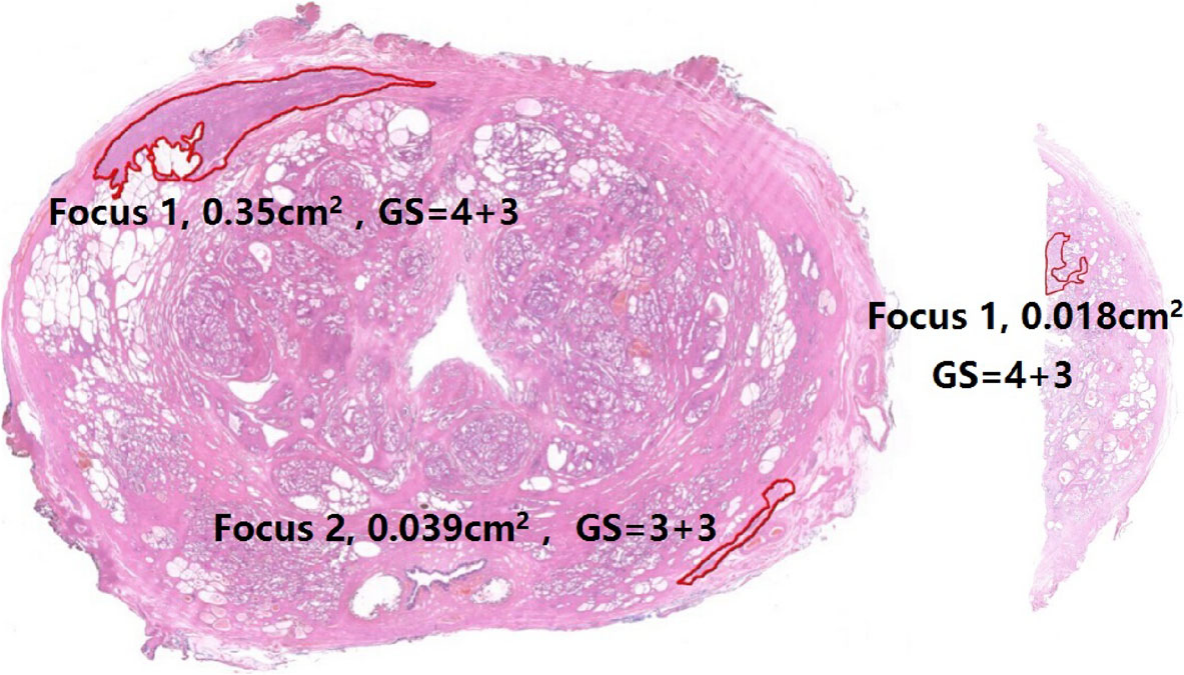
The positive region by biopsy was region 1. The whole-mount slice (left side) exhibited two foci, and the small slice (right side) had one focus (extension of focus 1 at the apex). The contours of all foci are outlined in red

The pathological slices were examined by a single pathologist with 14 years of experience and scanned into digital slices using digital scan equipment (NanoZoomer 2.0-RS, Photon, Japan). On the digital slices, we outlined tumor contours, calculated the tumor’s surface area, and determined the Gleason score (Fig. 2). The tumor volume was calculated by multiplying the tumor surface area by the thickness of the slice (0.4 cm of whole-mount slice or 0.2 cm of small slice) and then multiplying by a correction factor of 1.12 [9]. A specimen was defined as surgical margin positive if tumor cell invasion was microscopically observed on the ink-labeled prostate sections [10].

### Statistical methods

If normally distributed, data were described by the mean ± standard deviation (SD). If not normally distributed, data were described by the median and interquartile range (IQR). Chi-squared tests were used to compare the rates. SPSS 19.0 (Chicago, IL, USA) was used to analyze the data. A two-tailed test with *P* < 0.05 was considered statistically significant.

## Results

### General characteristics

The median age of the patients was 65 (IQR: 61–69) years old; the median serum prostate specific antigen was 11.7 ng/ml (IQR: 7.0–18.3 ng/ml), and the median prostate volume was 33.0 ml (IQR: 26.0–43.0 ml). Among the 171 individuals enrolled in this study, 56 (33%) received intravenous anesthesia, and 115 (67%) received local anesthesia.

### Tumor spatial distribution in biopsy specimens

Biopsy was implemented in 11 regions. One to four cores were obtained from each region, and the median total number of cores per patient was 24 (IQR: 22–28 cores). The median Gleason score was 7 (IQR: 6–7). The clinical staging was T1c–T3a (Table 1). The positive rates of biopsied regions 1–11 were 30, 28, 29, 30, 26, 27, 29, 23, 29, 31, and 43%, respectively. The positive rates did not significantly differ among regions 1–10 (χ2 = 4.15, *P* = 0.90). However, the mean positive rate of the 10 regions, 28%, was significantly lower than the positive rate in the apex (region 11) (χ2 = 8.61, *P* < 0.01).

**Table 1:** Comparison of the Gleason score and T-staging between prostate biopsy and radical prostatectomy specimens

	Biopsy specimens (*n* = 171)	Radical prostatectomy specimens (*n* = 171)
Gleason score, no. (%)		
6	62 (36)	28 (16)
3 + 4	65 (38)	81 (47)
4 + 3	21 (12)	38 (22)
8	14 (8)	8 (5)
9	9 (5)	16 (9)
T-staging, no. (%)		
T1c	86 (50)	
T2a	22 (13)	21 (1)
T2b	16 (9)	11 (6)
T2c	41 (24)	100 (59)
T3a	6 (4)	27 (16)
T3b	0 (0)	12 (7)

### Tumor spatial distribution in radical prostatectomy specimens

**Fig. 3:**
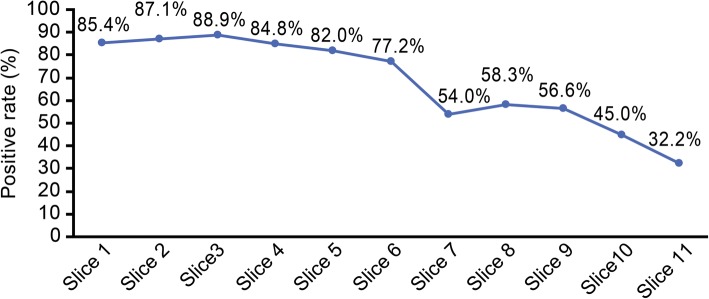
Diagram showing the positive rates of various longitudinal slices. Slice 1 = apex; slice 2 = apex above 0.5–0.9 cm; slice 3 = apex above 0.9–1.3 cm; slice 4 = apex above 1.3–1.7 cm; slice 5 = apex above 1.7–2.1 cm; slice 6 = middle layer; slice 7 = base below 2.2–2.6 cm; slice 8 = base below 1.8–2.2 cm; slice 9 = base below 1.4–1.8 cm; slice 10 = base below 1.0–1.4 cm; slice 11 = base

For 171 radical prostatectomy specimens, the median tumor volume was 1.5 ml (IQR 0.8–3.6 ml) and the median Gleason score was 7 (IQR: 7–7). The pathological staging was pT2a–pT3b (Table 1), and samples from 45 patients exhibited positive margins (26%). On the basis of the cutting method in radical prostatectomy specimens, the positive rates of various longitudinal slices are shown in Fig. 3. The positive rate was highest at the region 0.9–1.3 cm above the apex (89%), and it had a tendency to decrease towards the base. There was a significant difference in the positive rate between the cephalic (superior) and caudal (inferior) halves of the prostate (68% vs 99%, *P* < 0.01).

### Comparison between biopsy and radical prostatectomy specimens

There were no significant differences between the anterior and posterior zones in both the biopsy (71%vs 61%, *P* = 0.17) and radical prostatectomy (93%vs 87%, *P* = 0.08) specimens. Similarly, there were no significant differences in the tumor positive rates between the left and right sides of the prostate in biopsy (66%vs 68%, *P* = 0.65) and radical prostatectomy (92%vs 93%, *P* = 0.68) specimens.

Of the 171 patients, 460 lesions were detected, 191 of which were missed (42%). There were 107 (56%), 84 (44%), 105 (55%) and 86 (45%) missed lesions lying in the anterior, posterior, left and right sides, respectively. Of the 171 index lesions, 24 (14%) were missed, including 14 (58%), 10 (42%), 12 (50%) and 12 (50%) in the anterior, posterior, left and right sides, respectively.

Compared with the biopsy-derived Gleason score, the radical prostatectomy-derived Gleason score was unchanged, upgraded, and downgraded in 121 (71%), 43 (25%), and 7 (4%) patients, respectively. In addition, samples from 143 subjects (84%) had a Gleason score ≥ 3 + 4.

## Discussion

The accuracies of the prostate biopsy and tumor spatial distributions are important references for focal therapy. Template-guided transperineal saturation biopsy has advantages in tumor detection and localization and is generally considered as the preoperative assessment of focal therapy. Therefore, it is of great significance to specify the spatial distribution of prostate cancer on radical prostatectomy specimens diagnosed by template-guided transperineal saturation biopsy.

Although the long-term outcome of focal therapy remains to be established, it may achieve satisfactory short- to medium-term efficacy for patients with early-stage prostate cancer [11–13]. However, precise biopsy methods, which accurately assess tumor location and size, are needed for this treatment approach [1, 14]. To date, generally accepted methods include magnetic resonance imaging (MRI)-targeted and full transperineal template- mapping prostate biopsies [1]. Correspondingly, the type of focal therapy depends on the tumor location. Brachytherapy is appropriate for apical cancers to achieve less sphincter damage [15]. Given the shorter focal distance and more precise contouring of the target area, high-intensity focused ultrasound has obvious advantages for posterior tumors, and cryotherapy is suitable for anterior tumors to establish the oncologic efficacy [15]. Our results indicated that there was a good consistency in tumor spatial distribution between biopsy and radical prostatectomy specimens, which has a certain reference value.

Our study revealed the following characteristics in tumor spatial distribution diagnosed by template-guided transperineal saturation biopsy: (1) no apparent differences between the anterior and posterior zones or between the left and right sides, (2) the apex above the 0.9–1.3 cm portion of the gland had the highest positive rate, and (3) the positive rate of the upper half of the prostate was higher than that of the lower half. These findings are consistent with the results of Breslow et al. [16], who studied 1327 autopsy results from seven centers worldwide and discovered 350 cases of latent prostate cancer. Their analyses revealed that the tumor incidences were comparable between the anterior and posterior zones and between left and right sides and that the tumor incidence was significantly higher in the apex than in the basal aspect of the prostate with a tumor hotspot in the 0.5–1.5 cm zone next to the apex. The spatial distribution of the tumors revealed by the autopsy results indicated the original positions of the tumors, which could be employed to assess the ability of post-prostatectomy pathology to represent the actual tumor status and, further, to validate the accuracy of prostate biopsies. In general, with regard to the spatial distribution of the tumors, the biopsy results matched the radical prostatectomy results well (left and right sides, anterior and posterior zones, and upper and lower halves), which also agreed with the autopsy-based findings [16]. These results not only indicated the accuracy of template-guided transperineal saturation biopsy but also suggested that equal attention should be paid to the anterior and posterior zones. Vertically, the crucial biopsy area should be the apex and its vicinity. The matched tumor incidences between the left and right sides might be associated with the fact that the prostate is a left-right symmetrical organ. In addition, some studies have reported the tumor incidences between the anterior and posterior zones. For patients whose transrectal biopsies were previously found to be negative for tumors, a subsequent transperineal biopsy may identify tumors in the anterior zone (especially the anterior apical part of the prostate), where tumor incidence is significantly higher than that in the posterior zone [17]. These results indicate that transrectal biopsy may not identify tumors in the anterior zone and the apex, which is likely to be sampled by transperineal biopsy, as consistently reported in recent years [3]. Correspondingly, radical prostatectomy pathology revealed that patients who were previously diagnosed with prostate cancer via transrectal biopsy harbored significantly fewer tumors in the anterior zone than in the posterior zone because the transrectal approach failed to identify a considerable number of tumors in the anterior zone [17–19]. In our study, radical prostatectomy pathology revealed that the anterior zone did not harbor fewer tumors than the posterior zone, which is consistent with the tumor spatial distribution suggested by the autopsy study [16]. These results indicate that transperineal biopsy is superior to the transrectal approach for identifying anterior tumors. The highest positive rate of tumor was found in the apex in biopsy pathology, which can be explained such that the apex-neighboring tissues can be sampled when the apex is biopsied with a biopsy needle with a length of 1.8 cm. Therefore, the biopsy pathology was comparable to the radical prostatectomy pathology in this region. Notably, the autopsy-based prostate tumor incidence reported by Kido et al. [20] was primarily for the basal region and contradicted the findings by Breslow et al. [16]; however, Kido et al. [20] had a small sample size (*n* = 24) and only compared the tumor distributions in two layers.

In our study, a majority of patients (71%) had a Gleason score in biopsy consistent with the Gleason score in radical prostatectomy specimens. And those with Gleason score underestimated in biopsy accounted for about one fourth of the total patients. These results were in line with that of Huo’s study (65.7% unchanged, 25.6% upgraded and 8.8% downgraded) [21]. In Crawford’s study, patients with lower Gleason score in biopsy compared with radical prostatectomy specimens were equivalent to those with higher Gleason score (12% vs 16%) [7].

This study is subject to several limitations. First, MRI was not included as a necessary test in the design of the study. As a consequence, no comparison was attempted between MRI, biopsy pathology, and radical prostatectomy pathology. MRI is of vital importance for locating tumors and detecting major and clinically significant tumors [22–25]. Second, the 11-region biopsy was not adequately meticulous, and additional subdivisions (e.g., basal region and mid-region) should be employed. In addition, inking for localization was not performed for biopsied specimens; thus, their tumor spatial distribution was not precise. Third, the data were retrospectively analyzed; therefore, selection bias was unavoidable.Finally, we did not know the spatial distribution of actual prostate cancer lesions and had to resort to autopsy results as the gold standard, but the relevant autopsy studies were either obsolete or had a small sample size [16, 20].

## Conclusions

Our results revealed that the spatial distribution of the tumors generated by template-guided transperineal saturation biopsy were consistent with that of radical prostatectomy specimens in general. The tumor incidence rate was consistent between the anterior and posterior zones, and tumors in these two regions should be treated equally. The caudal (inferior) half of the prostate, especially the apex, was was the frequently occurred site of the tumor.

## Abbreviations

IQR: Interquartile range
MRI: Magnetic resonance imaging
SD: Standard deviation
